# Hantavirus Cluster Linked to Cruise Ship Travel: A Comprehensive Review of a Looming Global Public Health Concern

**DOI:** 10.7759/cureus.112246

**Published:** 2026-07-07

**Authors:** Venkata Bharat Kumar Pinnelli, Surendra Babu T, Jayashankar CA, Venkataramana Kandi, Mansi Thipani Madhu, Koshy T Sam, Manish GR, Divyadharshini MS, Atul S Sucharitha, Praful Bhupathiraju, Vinay S

**Affiliations:** 1 Biochemistry, Vydehi Institute of Medical Sciences and Research Center, Bengaluru, IND; 2 Anatomy, Vydehi Institute of Medical Sciences and Research Center, Bengaluru, IND; 3 Internal Medicine, Vydehi Institute of Medical Sciences and Research Center, Bengaluru, IND; 4 Clinical Microbiology, Prathima Institute of Medical Sciences, Karimnagar, IND; 5 Medicine, Vydehi Institute of Medical Sciences and Research Center, Bengaluru, IND; 6 General Medicine, Akash Institute of Medical Sciences and Research Center, Bengaluru, IND; 7 General Medicine, Vydehi Institute of Medical Sciences and Research Center, Bengaluru, IND; 8 Internal Medicine, Manipal Hospital, Bengaluru, IND

**Keywords:** andes virus (andv), diagnostics, epidemiology, hantaviruses, mv hondius expedition cruise ship, one health surveillance, risk identification, transmission

## Abstract

Hantaviruses are globally distributed, rodent-borne orthohantaviruses that cause hemorrhagic fever with renal syndrome in Eurasia and hantavirus cardiopulmonary syndrome (hantavirus pulmonary syndrome) in the Americas. Human infection usually results from environmental spillover through inhalation of aerosolized rodent excreta. However, the 2026 Andes virus (ANDV) cluster linked to the MV Hondius expedition cruise ship demonstrated how international travel networks can transform a rare zoonosis into a complex public health challenge. Following the Scale for the Assessment of Narrative Review Articles framework, this review synthesizes literature from 2019 to 2026 using explicit Boolean search strategies ("hantavirus" OR "orthohantavirus" OR "Andes virus" OR "ANDV") AND ("cruise" OR "maritime health" OR "shipboard medicine" OR "travel medicine" OR "outbreak response" OR "One Health") to evaluate global health governance and maritime outbreak preparedness. Unlike other human-pathogenic hantaviruses, ANDV is uniquely capable of limited person‑to‑person transmission through close, prolonged contact. Epidemiological investigation of the 2026 cluster confirmed that index cases were exposed during pre-embarkation terrestrial travel in highly endemic rural regions of Argentina and Chile, later manifesting on board. Clinically, hantavirus infection is characterized by severe endothelial dysfunction, capillary leak, and abrupt cardiopulmonary or renal failure following a nonspecific febrile prodrome. In the absence of curative antivirals or licensed vaccines, management depends on early risk recognition supported by a tiered diagnostic framework: rapid field-ready complete blood counts detecting thrombocytopenia and hemoconcentration to trigger stabilization and evacuation, followed by definitive molecular confirmation at centralized reference laboratories. The MV Hondius incident thus represents an operational biosecurity stress test rather than a pandemic threat, underscoring the need for integrated One Health surveillance, standardized maritime health governance, and legally codified protections for crew mental health and labor equity during isolation.

## Introduction and background

Hantaviruses are enveloped, negative‑sense ribonucleic acid (RNA) viruses with a segmented genome, classified within the family Hantaviridae and the order Bunyavirales. Their public health importance reflects a paradox: although transmission typically occurs at restricted ecological interfaces involving persistently infected rodents, severe infections can demand advanced intensive care, generate considerable public concern, and be readily disseminated through modern travel. Old world hantaviruses, such as Hantaan virus and others, including Seoul, Puumala, and Dobrava-Belgrade viruses, are primarily associated with hemorrhagic fever with renal syndrome (HFRS), whereas new world hantaviruses, such as Sin Nombre virus (SNV) and Andes virus (ANDV), cause hantavirus pulmonary syndrome (HPS) or hantavirus cardiopulmonary syndrome (HCPS) [1-5]. Although these syndromic categories are essential for clinical detection, they should not obscure the common pathogenic theme of vascular dysfunction, immunological activation, thrombocytopenia, capillary leak, and sudden clinical deterioration after an apparently vague febrile illness [6-8]. The epidemiology of hantavirus infection is best viewed through a One Health lens. Each medically significant hantavirus is maintained in one or more reservoir hosts, whose abundance, infection prevalence, behavior, and proximity to humans are influenced by food availability, rainfall, temperature, land conversion, living conditions, occupational activity, and recreational exposure [1,9-11]. Human infection is usually caused by inhaling aerosolized rat excreta; however, the risk is rarely evenly distributed throughout communities. Rural people, agricultural and forestry workers, cleaners of rodent-infested areas, military personnel, ecotourism professionals, and travelers accessing rodent habitats may be more exposed than the general population [2,12,13]. These ecological and social variables make hantavirus disease environmental, occupational, clinical, and political.

Among human‑pathogenic hantaviruses, ANDV remains unique as the only strain with repeatedly documented, albeit limited, person‑to‑person transmission. Epidemiological evidence from Argentina and Chile indicates that such transmission occurs predominantly in settings of close, sustained interpersonal contact, household, caregiving, or nosocomial, rather than casual community exposure [14-18]. This distinction is pivotal for public health policy: exaggerating interpersonal risk may prompt coercive measures, stigmatization, and erosion of trust, whereas underestimating it risks missed opportunities for prevention, contact monitoring, and early case recognition. The challenge is not merely to establish whether human transmission occurs, but to delineate its timing, frequency, routes, and contextual conditions of exposure. The 2026 outbreak linked to the expedition cruise ship MV Hondius exemplified how a rare zoonosis can be amplified through global travel networks. On May 2, 2026, the World Health Organization (WHO) issued an initial notification, followed by serial Disease Outbreak News updates; by May 13, WHO reported 11 cases, including three fatalities, with eight laboratory‑confirmed infections, two probable cases, and one inconclusive result pending further testing [19]. WHO assessed global public risk as low, prioritizing contact tracing, clinical care, dignified management of passengers and crew, diagnostic support, and International Health Regulations (IHR) coordination [20-22]. The episode was significant not because it suggested pandemic‑level transmissibility, but because it combined a severe zoonotic infection with delayed, nonspecific prodromes, mobile international contacts, constrained shipboard medical capacity, multijurisdictional oversight, and heightened post-coronavirus disease 2019 (COVID-19) public sensitivity to outbreak reporting.

Cruise and expedition vessels represent complex epidemic environments. They carry multinational passengers and crew, traverse shifting legal and public health jurisdictions, and often operate far from tertiary medical facilities. Shipboard medical teams must therefore make decisions under conditions of limited diagnostics, constrained isolation capacity, restricted laboratory support, and uncertain evacuation logistics. The social architecture of a vessel, shared cabins, communal dining, excursions, caregiving, and common services facilitates frequent interpersonal contact. These dynamics are well recognized in outbreaks of respiratory, gastrointestinal, foodborne, and vaccine‑preventable diseases; however, rare rodent‑borne zoonoses are seldom incorporated into maritime preparedness exercises [22,23]. The MV Hondius cluster thus expands the scope of cruise health beyond routine onboard outbreaks to encompass planning for rare, high‑impact infections. The post‑COVID‑19 context amplifies the importance of this incident. The pandemic exposed both strengths and vulnerabilities in surveillance, border control, laboratory capacity, health communication, quarantine ethics, supply chains, and international collaboration [24,25]. Yet applying COVID‑19 assumptions directly to hantavirus would be misleading. Unlike SARS‑CoV‑2, hantaviruses are not sustained by efficient respiratory transmission; their ecology is rodent‑associated, and ANDV human‑to‑human spread appears restricted to specific close‑contact scenarios. At the same time, COVID‑19 demonstrated how even small outbreaks can be socially magnified when uncertainty, fatalities, travel restrictions, and conflicting information converge. Preparedness must therefore adopt the governance principles refined during COVID‑19 while avoiding inaccurate epidemiological analogies.

This review builds on the editorial premise of a cruise‑associated hantavirus cluster and presents a comprehensive narrative synthesis to advance academic understanding. It integrates perspectives from virology, molecular biology, reservoir ecology, epidemiology, clinical medicine, diagnostics, medical countermeasures, One Health prevention, maritime health governance, policy analysis, lessons from COVID‑19, and future research priorities. The central argument is that the MV Hondius incident should be interpreted not as evidence of pandemic‑level transmissibility but as a systemic stress test: a test of whether clinicians can recognize rare infections early, laboratories can confirm diagnoses promptly, public health agencies can manage contacts proportionately, ship operators can safeguard passengers and crew humanely, and international systems can coordinate effectively before fear outpaces evidence.

## Review

Methodology

This narrative review examines hantavirus infection in the context of international travel, with particular reference to the 2026 MV Hondius ANDV cluster. A narrative design was adopted because the subject cannot be addressed through a single epidemiological question or pooled estimate and instead requires integration across diverse domains including mechanistic virology, reservoir ecology, clinical medicine, diagnostics, infection prevention, maritime operations, risk communication, ethics, and international health governance. The MV Hondius event is treated as an illustrative stress test rather than an evidence of a new epidemiological paradigm; the aim is not to suggest that cruise‑associated hantavirus events are common or that ANDV behaves as a pandemic respiratory virus but rather to explore what a rare, severe zoonotic cluster reveals about preparedness under conditions of uncertain exposure histories, cross‑border patient movement, delayed diagnostic confirmation, and public interpretation shaped by post‑COVID‑19 expectations. Information sources included PubMed‑indexed literature, clinical reviews, outbreak investigations, molecular studies, ecological analyses, diagnostic evaluations, and countermeasure studies, alongside contemporaneous official guidance from the WHO, the Pan American Health Organization, the European Centre for Disease Prevention and Control, and the United States Centers for Disease Control and Prevention [13,19,21,22,26,27]. Search themes encompassed hantavirus transmission mechanisms, reservoir ecology, clinical management, diagnostic workflows, and maritime public health regulations. The literature search was executed using explicit Boolean string combinations to ensure comprehensive retrieval, including: ("hantavirus" OR "orthohantavirus" OR "Andes virus" OR "ANDV") AND ("cruise" OR "maritime health" OR "shipboard medicine" OR "travel medicine" OR "outbreak response" OR "One Health"). Additional targeted searches paired primary pathogen terms with specific operational concepts, such as ("hantavirus pulmonary syndrome" OR "hantavirus cardiopulmonary syndrome") AND ("triage algorithm" OR "biomarkers" OR "quarantine ethics" OR "labor equity"). Priority was given to publications from 2019 to 2026 while retaining landmark molecular and epidemiological studies for foundational context [1,6,28-31]. Eligibility criteria encompassed sources addressing hantavirus biology, ecology, epidemiology, transmission, clinical features, diagnostics, infection control, countermeasures, surveillance, maritime governance, or preparedness lessons; sources unrelated to viral hemorrhagic fevers or to general travel medicine advice were excluded. Evidence was synthesized thematically, progressing from biological and ecological foundations to clinical recognition, diagnostics, management, and policy, reflecting the practical sequence of events in outbreak response. Throughout, the review distinguishes established evidence (e.g., rodent ecology, cardiopulmonary syndrome, limited person‑to‑person transmission of ANDV), plausible inference (e.g., relevance of cruise ship density and mobility to contact management), and unresolved uncertainty (e.g., relative importance of transmission routes, frequency of mild infection, and thresholds for critical‑care transfer).

Application of the Scale for the Assessment of Narrative Review Articles Framework

To ensure methodological transparency and structural rigor, this narrative review was formally appraised using the Scale for the Assessment of Narrative Review Articles framework [32]. The manuscript was systematically evaluated across all six domains: justification of relevance, articulation of concrete operational aims, completeness of the literature search strategy, adequacy of peer‑reviewed referencing, clarity of scientific reasoning, and appropriate presentation of categorical and visual data. Each domain was scored on the standard 0-2 scale (0 = low quality/missing; 1 = intermediate; 2 = high quality), thereby minimizing selective reporting bias, upholding rigorous standards of scientific reporting, and delivering an objective, actionable synthesis of travel‑associated public health threats (Table 1).

**Table 1:** Quality assessment of the narrative review article using the SANRA framework CDC: Centers for Disease Control and Prevention; ECDC: European Centre for Disease Prevention and Control; PAHO: Pan American Health Organization; SANRA: Scale for the Assessment of Narrative Review Articles; WHO: World Health Organization

SANRA domain	Description and evaluation metric	Assessment/application in this review	Score for each domain (0-2); total score = 12
Justification of the item's importance	Explicitly explains why the review is important and relevant to the readership	Heavily justified in the introduction by highlighting the public health paradox of rare zoonoses intersecting with modern international travel networks	2
Statement of concrete aims	Formulates specific operational objectives or primary questions addressed by the review	Aims are concretely stated to evaluate global health governance, maritime outbreak preparedness, and operational biosecurity stress-testing	2
Description of literature search	Details the search strategy, including databases, explicit Boolean terms, and inclusion/exclusion criteria	Comprehensively outlined using explicit Boolean string combinations, specific paired operational search themes, and clear eligibility bounds	2
Referencing	Ensures statements are supported by appropriate, accurate, and peer-reviewed citations	Backed by robust tracking of landmark molecular studies alongside contemporary public health institutional alerts (WHO, CDC, PAHO, ECDC)	2
Scientific reasoning	Presents arguments clearly, progressing logically from evidence to conclusions	Chronologically tracks evidence moving from biological foundations to clinical data triage, real-world cohort tracking, and ethical policy logic	2
Presentation of data	Appropriate use of clear, categorical, tabular, and visual data representations	Structured data presentation utilizing clinical matrices, outbreak timelines, and multitiered life cycle/pathogenesis schematics	2
Total score	Maximum possible score: 12	High-quality scientific narrative review	12

Classification and molecular biology

**Figure 1:**
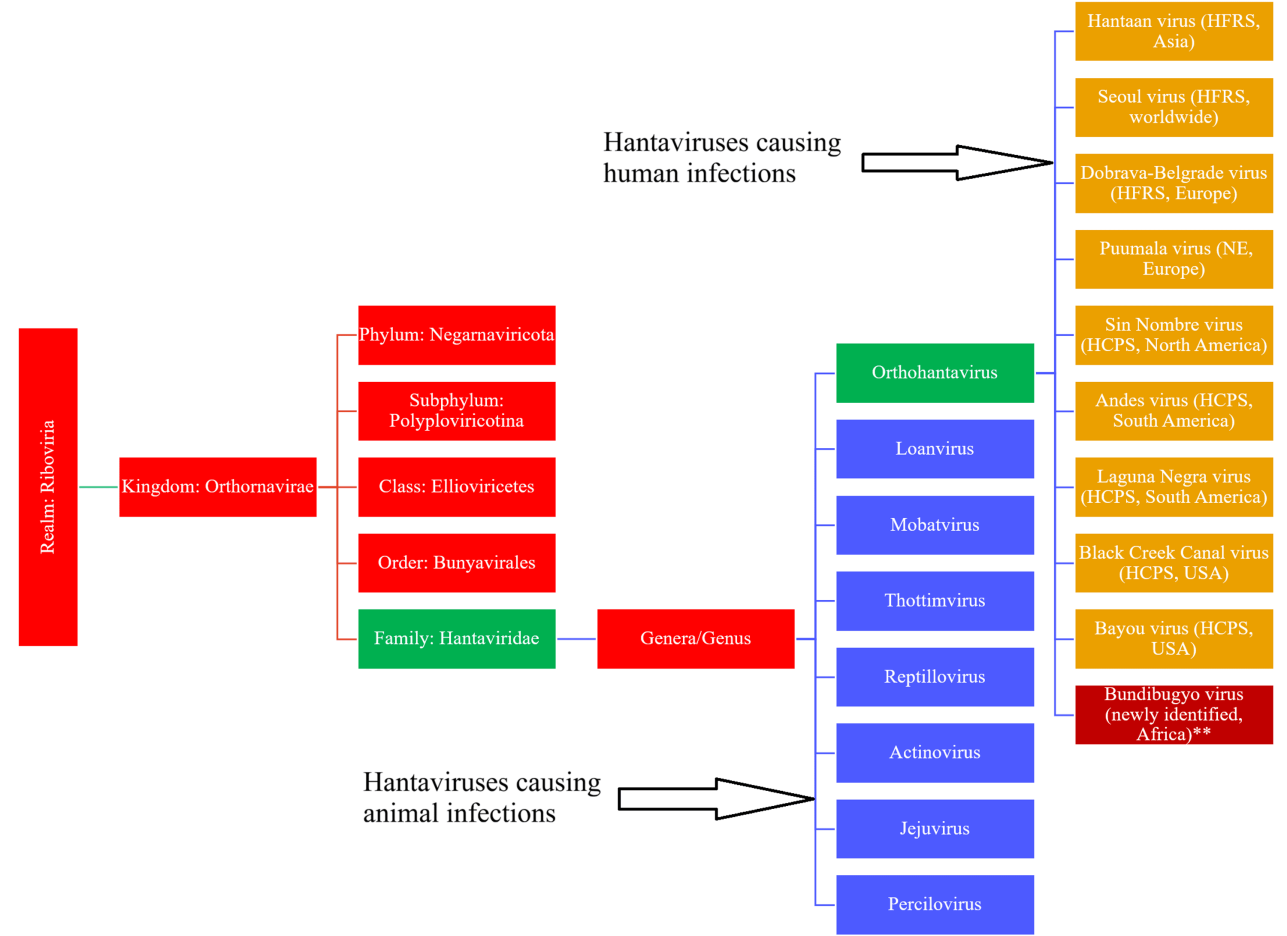
Taxonomical position of hantaviruses ^**^The newly identified Bundibugyo variant HFRS: hemorrhagic fever with renal syndrome; HCPS: hantavirus cardiopulmonary syndrome; HPS: hantavirus pulmonary syndrome; NE: nephropathia epidemica Image credit: This is an original image created by the author Venkataramana Kandi using Microsoft Word/PowerPoint (Microsoft® Corp., Redmond, WA)

The classification of hantaviruses has evolved significantly with advances in molecular virology and phylogenetic analysis, leading to a clearer understanding of their taxonomic organization within the family Hantaviridae and order Bunyavirales [30,33-36]. Figure 1 provides a visual overview of this classification framework, highlighting the hierarchical structure of hantaviruses and their placement among related viral taxa. This schematic serves as a foundation for subsequent discussion on geographic distribution, host reservoirs, and clinical syndromes, offering readers a concise reference point for the complex taxonomy of these emerging pathogens [1,6,12] (Figure 1).

Hantaviruses are enveloped, negative‑sense RNA viruses with a tripartite genome composed of three segments: the small (S) segment, which encodes the nucleocapsid (N) protein essential for RNA encapsidation and modulation of host immune responses; the medium (M) segment, which encodes a glycoprotein precursor subsequently cleaved into Gn and Gc, mediating viral attachment, entry, and fusion through interactions with host cell receptors such as β3 integrins and heparan sulfate proteoglycans; and the large (L) segment, which encodes the viral RNA‑dependent RNA polymerase (RdRp) responsible for transcription and replication of viral RNA [30,33-36]. Advances in high‑throughput sequencing have broadened hantavirus taxonomy, identifying hantavirus‑like viruses across diverse hosts including rodents, shrews, moles, bats, reptiles, and fish [5,30,31]. Despite this phylogenetic diversity, the clinically relevant distinction remains pragmatic: Old World hantaviruses are classically associated with HFRS, whereas New World hantaviruses are linked to HCPS/HPS. Although this dichotomy is imperfect due to overlapping clinical features, it remains useful for diagnosis, risk communication, and surveillance [1,6,12].

The ANDV, endemic in Argentina and Chile, is maintained in its principal rodent reservoir, the long‑tailed pygmy rice rat (*Oligoryzomys longicaudatus*) [37,38]. Molecular analyses highlight ANDV’s unique capacity among human‑pathogenic hantaviruses for limited person‑to‑person transmission, particularly in contexts of intimate contact, caregiving, and nosocomial exposure [14-17]. This transmissibility is thought to be influenced by viral load dynamics, glycoprotein‑mediated cell tropism, and host immune modulation, underscoring the need for integrated molecular, ecological, and clinical perspectives in outbreak preparedness. To provide a clear etiological framework, the formal taxonomic architecture, geographic distribution, and primary clinical presentations of human-pathogenic hantaviruses are categorized below, with primary pathogens summarized in Table 2.

**Table 2:** Global syndromic matrix, reservoir ecology, and transmission profiles of the order Bunyavirales (family Hantaviridae) HFRS: hemorrhagic fever with renal syndrome; HPS: hantavirus pulmonary syndrome; HCPS: hantavirus cardiopulmonary syndrome; ANDV: Andes virus; SNV: Sin Nombre virus; HTNV: Hantaan virus; PUUV: Puumala virus Source: This table has been synthesized by the authors based on the data from [14-17,37,38]

Virus	Geographic group	Primary reservoir host	Major clinical syndrome	Human interpersonal spread
Hantaan (HTNV)	Old World (Asia/Europe)	Rodents (Apodemus spp.)	HFRS (kidney targeting)	No
Puumala (PUUV)	Old World (Europe)	Bank voles (Myodes glareolus)	Mild HFRS/nephropathia epidemica	No
Sin Nombre (SNV)	New World (North America)	Deer mice (Peromyscus maniculatus)	HPS/HCPS (Lung targeting)	No
Andes (ANDV)	New World (South America)	Long-tailed pygmy rice rat (Oligoryzomys longicaudatus)	HPS/HCPS (Severe cardiopulmonary)	Yes (limited to close/sustained contact)

Mechanistic Description of the Hantavirus Life Cycle

Human‑pathogenic hantaviruses persist ecologically in asymptomatically infected rodent reservoirs, such as *Oligoryzomys longicaudatus* for ANDV, which chronically shed virus into the environment via saliva, urine, and feces. Human infection occurs predominantly through inhalation of aerosolized excreta, after which the virus undergoes a defined intracellular cycle. Entry begins with viral envelope glycoproteins (Gn/Gc) binding to host receptors, followed by receptor‑mediated endocytosis into clathrin‑coated endosomes. Acidification within endosomes triggers membrane fusion and uncoating, releasing viral ribonucleoprotein complexes into the cytoplasm. The viral RdRp then transcribes and replicates the three negative‑sense genomic segments, while host ribosomes translate viral messenger RNAs into nucleocapsid, glycoprotein, and polymerase proteins. Assembly occurs at Golgi‑derived membranes, where nucleocapsid‑bound RNA segments interact with glycoproteins to form mature virions. These particles are subsequently released via budding and exocytosis, completing the replication cycle and enabling further spread within the host.

Reservoir ecology and One Health drivers

Human hantavirus infection is usually a spillover event after inhalation of aerosolized rodent urine, feces, or saliva, with less frequent infection through bites or direct contamination [1,3,13]. The specificity of virus-host relationships means that human risk changes with the abundance, infection prevalence, movement, and human proximity of reservoir species. Ecological fluctuations are influenced by rainfall, mast events, temperature, food availability, predators, land conversion, agricultural activity, housing quality, and occupational or recreational exposure [1,9-11]. Recent systematic evidence reinforces that land cover and land use are not background variables; they are drivers of exposure. A systematic review of land-cover and land-use associations found heterogeneous but recurring links between human infection risk and agricultural, forest-edge, and disturbed landscapes [10]. Climate-focused evidence from Latin America and the Caribbean similarly suggests that rainfall, humidity, vegetation, and temperature may shape rodent dynamics and human exposure, although models vary across regions and reservoirs [11,39]. Occupational exposure remains important. Agricultural workers, forestry workers, military personnel, cleaners of rodent-contaminated spaces, ecotourism workers, and residents of rodent-infested dwellings have higher exposure potential [2,12]. Expedition tourism adds an additional layer: travelers may enter high-risk rural or wilderness environments and then rejoin dense transport settings before incubation is complete. The cruise setting, therefore, need not be the original ecological exposure site to become an outbreak-management setting.

Epidemiology and global burden

Hantavirus burden is unevenly distributed and incompletely measured. HFRS accounts for the largest global case numbers, particularly in East Asia and parts of Europe [1,4,12]. HCPS/HPS is less common but has a higher case-fatality range, often reported around 30%-40% for severe New World disease and higher in some historical clusters [2,6,37]. Seroprevalence data suggest that mild or undiagnosed infections occur, but estimates are heterogeneous because assays, populations, and exposure definitions differ [40,41]. In the Americas, HCPS/HPS surveillance depends on clinician suspicion, compatible exposure history, serology, molecular testing, and reporting systems. The 1993 Four Corners outbreak in the United States led to recognition of SNV as a cause of severe pulmonary disease and transformed hantavirus surveillance in North America [12,29,42]. In South America, ANDV and related genotypes produce sporadic cases, family clusters, and occupational or rural outbreaks, with Argentina, Chile, Brazil, Bolivia, Paraguay, and other countries contributing to the regional burden [38,41]. The MV Hondius event is best understood as a travel-associated ANDV cluster rather than as evidence of sustained global transmission. WHO’s May 13, 2026, update identified a multinational group of cases and deaths but also assessed the risk to the global population as low [19]. The public health significance lies in the difficulty of managing potential contacts across jurisdictions, not in a proven capacity for pandemic spread comparable to respiratory viruses such as severe acute respiratory syndrome coronavirus-2 (SARS-CoV-2).

To anchor the operational lessons of the MV Hondius incident in epidemiological reality, the outbreak chronology and clinical‑demographic profile must be precisely defined. Between April and May 2026, a total of 11 cases were recorded, including three fatalities, yielding a case‑fatality rate of 27.3%. Laboratory confirmation through qRT‑PCR and IgM serology identified ANDV in eight patients, while two were classified as probable epidemiological links and one remained inconclusive due to borderline viral load values. The cohort reflected the multinational, highly mobile demographic typical of polar expedition tourism, spanning five countries across Europe and the Americas, with a median age of 54 years (range, 31-72) and a slight male predominance (63.6%). Exposure stratification showed that the primary index cluster comprised international passengers who had undertaken extensive pre‑embarkation trekking in endemic Patagonian regions of Argentina and Chile, whereas secondary shipboard cases, including cabin mates and a healthcare provider delivering close clinical care, developed illness later, underscoring the context‑dependent nature of ANDV transmission. The reconstructed timeline revealed an average incubation period of 24 days (range, 12-39) from land‑based exposure to febrile prodrome onset. Clinically, all confirmed patients presented with abrupt fever (>38.5°C), severe myalgia, and profound malaise, with 72.7% reporting early gastrointestinal symptoms. Progression to the cardiopulmonary leak phase was rapid, occurring at a median of 4.5 days after symptom onset, and was marked by severe thrombocytopenia (median nadir 42,000/µL), hematocrit elevation >20% from baseline, and diffuse pulmonary infiltrates, necessitating urgent evacuation and intensive ventilatory or vasopressor support (Table 3) [43-46].

**Table 3:** Epidemiological, demographic, and clinical profile of the MV Hondius Andes virus outbreak cohort IgM: immunoglobulin M; qRT-PCR: quantitative reverse transcription polymerase chain reaction Source: This table has been synthesized by the authors based on the data from [43-46]

Parameter	Cohort metric/characteristic
Total outbreak size	11 cases
Laboratory confirmation status	8 confirmed (qRT-PCR and IgM positive), 2 probable (epidemiologically linked), 1 inconclusive
Outcomes and mortality	3 fatalities, 8 recoveries (Case-fatality rate: 27.3%)
Geographic/demographic origin	Multinational cohort spanning 5 distinct countries across Europe and the Americas
Age distribution	Median age: 54 years (range: 31-72 years)
Sex distribution	63.6% male, 36.4% female
Primary source of infection	Environmental spillover (inhalation of aerosolized rodent excreta from Oligoryzomys longicaudatus) during pre-embarkation terrestrial trekking in rural Chilean and Argentinean Patagonia wilderness sectors
Secondary source of infection	Limited, context-dependent person-to-person transmission onboard via close, prolonged contact (affecting cabin mates and a maritime healthcare provider)
Incubation period window	Average: 24 days (range: 12-39 days)
Initial clinical presentation	100% abrupt-onset fever (>38.5^°^C), severe myalgia, and profound malaise; 72.7% early gastrointestinal distress
Cardiopulmonary progression timeline	Median of 4.5 days from onset of prodromal symptoms to acute respiratory leak phase
Key laboratory and diagnostic findings	Severe thrombocytopenia (median platelet nadir: 42,000/μ, marked hemoconcentration (hematocrit elevation >20 from baseline), and diffuse bilateral pulmonary infiltrates
Critical care management requirements	Urgent medical evacuation from the vessel, intensive mechanical ventilatory support, or vasopressor therapy

Transmission dynamics

Environmental Transmission

For most hantaviruses, human infection follows environmental exposure rather than interpersonal spread. The central exposure pathway is inhalation of particles contaminated by urine, feces, or saliva from infected reservoir rodents. This can occur when people enter cabins, sheds, barns, storage rooms, military shelters, field stations, or poorly ventilated buildings where rodents have nested or fed. Disturbance is especially important: sweeping, vacuuming, moving stored materials, handling firewood, opening long-closed rooms, or cleaning without wetting surfaces can aerosolize infectious material and convert a localized contamination problem into an inhalational hazard [13,47]. Environmental transmission is also shaped by timing. Human risk may rise after ecological conditions increase rodent abundance, after seasonal behavior brings rodents indoors, or after human activities place people in rodent habitat. Rainfall, vegetation growth, food availability, land-use change, and housing quality can alter the probability that infected rodents and susceptible people share the same space [9-11]. These dynamics help explain why cases often appear as sporadic events or small clusters rather than as large waves of sustained human transmission. The prevention logic is, therefore, practical and environmental. Rodent exclusion, sealing entry points, safe food storage, waste control, ventilation, wet disinfection before cleaning, gloves and respiratory protection for high-risk cleanup, and avoidance of sleeping in rodent-infested structures are more important than interventions designed for efficient airborne human viruses [13,47]. In travel settings, these measures should be implemented before passengers arrive at rural lodges, expedition staging sites, storage areas, and excursion facilities. A cruise ship may become the location where illness is recognized, but the biologically relevant exposure may have occurred days or weeks earlier during shore-based travel.

Although a cruise vessel represents a tightly contained environment that can magnify operational challenges, the biological origins of a travel‑associated hantavirus outbreak are rooted in the distinct terrestrial ecologies visited before embarkation. This distinction is exemplified by the 2026 MV Hondius ANDV cluster. The ship departed Ushuaia, Argentina, for an itinerary across the South Atlantic and Antarctica, yet epidemiological investigations revealed that the index cases had undertaken extended land travel in rural, highly endemic regions of Argentina and Chile before reaching the port. This pre‑embarkation exposure period corresponds closely with the known one‑to‑six‑week incubation window of ANDV, indicating that spillover occurred in terrestrial landscapes rather than on board. In South American wilderness and rural ecotourism zones, human risk is shaped by microhabitats shared with the long‑tailed pygmy rice rat (*O. longicaudatus*). Trekking along disturbed forest edges, lodging in rustic wilderness facilities, or entering poorly ventilated storage spaces create prime conditions for inhaling aerosolized rodent excreta. Once infected individuals transition from these high‑risk environments into the dense social networks of an expedition ship, outbreak dynamics shift: environmental spillover gives way to the rare possibility of limited person‑to‑person transmission. Reconstructing this chain of exposure highlights that maritime preparedness must integrate land‑based zoonotic hazards as inseparable components of a traveler’s itinerary [43,45,46].

This approach prevents two errors: assuming that all cases must have been infected on board or dismissing shipboard management needs because the original exposure was likely on land. As demonstrated by the MV Hondius timeline, both environmental and interpersonal pathways must be critically assessed side by side until diagnostic data, serial viral sequencing, and detailed exposure histories definitively isolate the true primary source.

ANDV and Person-to-Person Transmission

ANDV is the major exception to the rule that hantaviruses are not transmitted between people. Molecular evidence from Argentina provided early support for person-to-person transmission, and subsequent investigations described household and nosocomial transmission [14,15,47,48]. A prospective household-contact study in Chile suggested close-contact risk, although later systematic assessment emphasized that much of the evidence base is observational and vulnerable to co-exposure bias [16,18]. This caveat matters because people who live, work, travel, or socialize together may also share environmental rodent exposures, making it difficult to separate direct human transmission from common-source infection. The epidemiological pattern of ANDV transmission is best described as limited, inefficient, and highly context-dependent. Reported transmission events have generally involved intimate partners, household members, caregivers, healthcare exposures, or prolonged close contact rather than brief casual encounters [16-18]. This pattern does not support a comparison with SARS-CoV-2 or influenza. It does, however, justify careful contact tracing, symptom monitoring, and infection prevention for people who had close exposure to an ill patient during the potentially infectious period. The 2018-2019 Epuyen outbreak in Chubut Province, Argentina, strengthened the evidence base by combining epidemiology and genomics. Martinez et al. reported 34 confirmed infections and 11 deaths, with transmission chains compatible with person-to-person spread and possible superspreading [17]. The outbreak was important because it showed how a rare zoonotic infection can create multiple generations of transmission under favorable social and biological conditions. It also showed the practical difficulty of defining exposure categories when community relationships, caregiving, social gatherings, and household contacts overlap. A later virological characterization of an Epuyen-related ANDV isolate provided a contemporary platform for countermeasure research and experimental work [49].

Biological plausibility has also increased. A prospective Chilean study detected ANDV RNA and infectious virus in body fluids, including gingival crevicular fluid, saliva, nasopharyngeal specimens, and urine during acute infection, and linked detection outside blood with disease severity [50]. Experimental modeling of human-to-human ANDV transmission in Syrian hamsters further supports investigation of respiratory and mucosal routes [51]. These findings do not prove that the body fluid detection event is epidemiologically important, but they narrow the gap between observed transmission and a plausible mechanism. They also reinforce the need for standard precautions, respiratory protection during high-risk clinical procedures, safe handling of body fluids, and careful management of close caregivers.

For the MV Hondius response, WHO adopted a precautionary contact-management approach. The interim guidance defined the suspected, probable, confirmed, and noncase categories; recognized a typical incubation period of one to six weeks; and recommended active monitoring and risk-based classification for contacts, including passengers and crew [21]. This stance is appropriate because transmission is limited and uncertain, but the consequences of missing severe disease can be high. The goal of contact management should not be to dramatize risk or imply pandemic potential; it should be to identify exposed people early enough for symptom monitoring, rapid clinical evaluation, laboratory testing, and supportive care.

Risk communication must therefore be precise. Saying that hantaviruses “do not spread between people” is too broad for ANDV and may delay recognition of close-contact risk. Saying that ANDV is “airborne like COVID-19” is also inaccurate and may provoke disproportionate restrictions. The most useful message is narrower: most hantavirus infections come from rodent-contaminated environments, while ANDV has occasionally spread between people after close and prolonged contact. That formulation supports targeted precautions without stigmatizing passengers, crew, households, or endemic communities.

Clinical spectrum and pathogenesis

Hantavirus infection begins when humans inhale aerosolized excreta, including urine, feces, and saliva, from chronically infected reservoir rodents [6,52]. The virus primarily targets endothelial cells lining blood vessels, with entry mediated by viral glycoproteins (Gn and Gc) binding to host receptors such as beta‑3 integrins [7]. Once internalized, hantaviruses replicate within the cytoplasm and activate host immune responses [8]. Cytokine release, including tumor necrosis factor‑alpha, interleukin (IL)‑1β, IL‑6, and interferon‑gamma, amplifies inflammation [53]. Endothelial activation, marked by upregulation of adhesion molecules such as intercellular adhesion molecule-1 and vascular cell adhesion molecule-1, disrupts vascular integrity [54]. The resulting capillary leak produces hemoconcentration, thrombocytopenia, and fluid extravasation [55]. Pathogenesis is dominated by dysregulated vascular permeability rather than direct cytolysis, with contributions from viral infection of endothelial cells, monocytes and macrophages, platelet activation, complement pathways, and angiopoietin signaling [7,8,53,54].

Clinically, this vascular dysfunction manifests as two overlapping syndromes. HFRS is characterized by renal involvement, hypotension, hemorrhagic features, and oliguria [4,6]. HPS/HCPS presents with abrupt progression from a nonspecific prodromal phase presenting with fever, myalgia, headache, chills, malaise, gastrointestinal symptoms, to cough, dyspnea, hypoxemia, pulmonary edema, shock, arrhythmias, and multiorgan failure [6,37,52]. Despite differences in predominant organ involvement, both syndromes share a common pathogenic basis in endothelial dysfunction and capillary leakage (Table 4) [7,55,56].

**Table 4:** Pathogenesis matrix of human pathogenic hantavirus infections HFRS: hemorrhagic fever with renal syndrome; HPS: hantavirus pulmonary syndrome; HCPS: hantavirus cardiopulmonary syndrome; TNF-α: tumor necrosis factor-alpha; IL-1β: interleukin-1 beta; IL-6: interleukin-6; IFN-γ: interferon-gamma Source: This table has been synthesized by the authors based on the data from [1,2,4,6-8,27,33,35,36,42,50,53-55,57,58]

Pathogenic phase	Underlying mechanisms and cellular targets	Clinical manifestations/syndrome
Initial spillover and cellular entry	Human infection initiates via the inhalation of aerosolized rodent excreta [1]. Viral envelope glycoproteins (Gn/Gc) bind to host cell surface receptors (e.g., β3 integrins, heparan sulfate proteoglycans), triggering receptor-mediated endocytosis into clathrin-coated endosomes [35,36]	Characterized by a nonspecific febrile prodrome: abrupt-onset fever (>38.5^°^C), severe myalgia, headache, chills, and profound malaise [6,27]
Endothelial and immune activation	The virus undergoes localized replication within endothelial cells, monocytes, and macrophages without causing direct cytolysis [33]. This induces robust cellular and innate immune activation, marked by a massive expansion of activated T-cells and complement pathways [53,54]	Early gastrointestinal distress (nausea, vomiting, diarrhea, abdominal pain), presenting in up to 72.7% of cases in travel-associated cohorts [6,27]
Vascular permeability and capillary leak	Disruption of the endothelial barrier function occurs through immune-mediated injury, platelet activation, and dysregulated angiopoietin signaling [8,55]. Hypercytokinemia (TNF-α, IL-1β, IL-6, IFN-γ) drives widespread endothelial dysfunction [7]	HCPS/HPS: profound capillary leak, hemoconcentration (hematocrit elevation >20%), severe thrombocytopenia, and diffuse bilateral pulmonary infiltrates causing acute hypoxia [42,50]
Systemic collapse and end-organ failure	Exuberant inflammatory cascades overwhelm host regulatory mechanisms, culminating in massive, uncontrolled fluid shifts into interstitial spaces, intravascular volume depletion, and profound cardiac or renal stress [57,58]	HCPS: cardiopulmonary shock, severe arrhythmias, and multiorgan failure requiring intensive mechanical ventilation or vasopressor therapy [2]. HFRS: severe renal dysfunction, systemic hypotension, oliguria, and hemorrhagic manifestations [4]

Host immunity has dual roles. Neutralizing antibodies (NAbs) are protective and associated with viral control, but exuberant cellular and inflammatory responses may worsen vascular leak [57,58]. In Argentinean HPS patients, delayed viral clearance occurred despite high numbers of activated thymus (T) derived lymphocytes or T-cells, supporting the view that humoral and innate responses, not T-cell activation alone, are central to recovery [58]. Clinical triage must recognize that early symptoms can mimic influenza, COVID-19, dengue, leptospirosis, bacterial sepsis, viral pneumonia, or gastroenteritis [6,12,37]. A travel and exposure history is therefore as important as the physical examination. In cruise-associated events, clinicians should ask about ship exposure, cabin contacts, caregiving, shared meals, port visits, wilderness or rodent exposure, cleaning activities, and contact with known cases.

Diagnosis and surveillance

Laboratory confirmation relies on serology, molecular testing, or immunohistochemistry. Immunoglobulin M (IgM) and rising immunoglobulin G (IgG) titers are widely used; reverse transcription-polymerase chain reaction (RT-PCR) can be valuable during acute illness, particularly for ANDV, when viral RNA may be detectable in blood and other specimens [12,50]. Because cross-reactivity and assay availability vary, reference laboratory support is essential, especially for rare, imported cases or multinational clusters.

Diagnostic surveillance should connect patient testing with environmental and rodent investigations. Following a confirmed case, public health teams should reconstruct likely exposure sites, capture reservoir rodents when appropriate and safe, sequence viral genomes from human and rodent samples, and compare sequences to infer source and transmission chains [12,49,59]. Genomic epidemiology cannot replace field epidemiology, but it can distinguish a shared environmental source from sequential transmission when enough high-quality samples are available.

The MV Hondius response illustrates the need for deployable diagnostic networks. WHO reported arranging shipment of diagnostic kits from Argentina to laboratories in five countries and coordinating expert calls across laboratory, clinical, epidemiological, and infection prevention domains [19,20]. A future-ready system would preidentify reference laboratories for rare zoonoses, validate specimen pathways, and maintain cross-border data-sharing agreements before crises occur.

Clinical management and medical countermeasures

Effective management of hantavirus disease begins with early recognition of nonspecific prodromal symptoms, such as fever, myalgia, malaise, and gastrointestinal upset, combined with a compatible travel or exposure history [3]. Laboratory triage relies on rapid complete blood counts, where thrombocytopenia and hemoconcentration serve as early warning signals of impending capillary leak [52]. Because molecular confirmation through RT‑PCR or serology is often delayed, initial stabilization must proceed on clinical suspicion [60]. Supportive care remains the cornerstone of treatment [3,52,60]. In HFRS, management emphasizes cautious fluid administration, monitoring for hypotension, renal replacement therapy when indicated, and control of hemorrhagic complications [4,6]. In HPS/HCPS, aggressive cardiopulmonary stabilization is required, including oxygen supplementation, mechanical ventilation (continuous positive airway pressure or bilevel positive airway pressure), vasopressor support, and, in severe cases, extracorporeal membrane oxygenation (ECMO) [37,61].

Pharmacologic interventions for hantavirus disease remain limited. Ribavirin has demonstrated benefit in HFRS when administered early, although its efficacy in HPS/HCPS is weaker, and issues of timing, toxicity, and route complicate its use [52,62-64]. Other agents, including favipiravir, polymerase inhibitors, monoclonal antibodies, and nucleic acid-based platforms, remain investigational or preclinical, with no licensed therapies currently available [60,64,65]. Passive immunotherapy and NAb strategies are biologically attractive for ANDV, as neutralizing responses correlate with viral clearance and protection. However, clinical evidence remains insufficient to support routine use outside of clinical trials or compassionate‑use frameworks [58,60]. Vaccine development has explored multiple platforms, including inactivated vaccines deployed in parts of Asia, deoxyribonucleic acid vaccines, viral‑vectored candidates, virus‑like particles, and subunit approaches [60,66,67]. Despite these advances, no globally licensed vaccine exists for HPS/HCPS, and the rarity of outbreaks complicates efficacy trials [60]. Future policy‑relevant pathways may require immunobridging, animal‑rule regulatory approaches, regional stockpiles for high‑risk groups, and coordinated trial protocols activated during outbreak clusters (Table 5) [19,60].

**Table 5:** Tiered diagnostic and management framework for hantavirus infections SpO₂: peripheral capillary oxygen saturation; HFRS: hemorrhagic fever with renal syndrome; IgG: immunoglobulin gamma; IgM: immunoglobulin mu; IL-6: interleukin-6 Source: This table has been synthesized by the authors based on the data from [1,6,12,17,26,37,42-44,50,52,59-61,63]

Care tier/setting	Diagnostic trigger and biomarkers	Clinical management protocol
Tier 1: field/remote settings, including shipboard infirmaries, rural clinics, and expedition posts	Clinical evaluation of compatible ecological exposure and recent travel history in endemic sectors [43,52]; serial complete blood count monitoring reveals a biphasic field signal: a sharp decline in platelets (median nadir 42,000 μL) concurrent with rising hematocrit (>20% from baseline) [43,44]	Strict, cautious fluid restriction to avoid exacerbating pulmonary vascular leakage [26,37]; immediate cardiorespiratory stabilization and close clinical observation [26,52]; execution of prepositioned, urgent medical evacuation arrangements to a tertiary intensive care center [43,52]
Tier 2: tertiary care centers including advanced intensive care units	Monitoring of rapid clinical progression from the nonspecific febrile prodrome into the acute respiratory leak phase [42]; continuous SpO_2_ tracking, arterial blood gas analysis, and serial chest radiography showing diffuse, bilateral pulmonary infiltrates [37,42]	Early escalation to noninvasive respiratory support or invasive mechanical ventilation [6,61]; targeted vasopressor therapy to combat cardiovascular shock and hemodynamic collapse [6,61]; deployment of extracorporeal membrane oxygenation in selected, refractory cardiopulmonary cases [6,61]
Tier 3: reference laboratories, including centralized confirmation and stratification centers	Definitive molecular confirmation via quantitative reverse transcription-polymerase chain reaction to track viral load dynamics in blood or tissue samples [1,50]. Serological tracking via enzyme-linked immunosorbent assays detecting hantavirus-specific IgM and rising IgG titers [1,12]; multiplex immunoassays for plasma biomarkers of endothelial distress (e.g., elevated IL-6, angiopoietin balance) [50,59]	Formal epidemiological case classification based on international health guidelines (suspected, probable, confirmed, vs. noncase) [68]; invalidation or confirmation of common-source vs. close-contact transmission chains using high-throughput genomic sequencing [17]; administration of early intravenous ribavirin for HFRS cases, or enrollment in investigational countermeasure trials (monoclonal antibodies, neutralizing passive immunotherapy, or novel polymerase inhibitors) [60,63]

The MV Hondius cluster as a preparedness stress test

The cruise-associated cluster exposes vulnerabilities that are not unique to hantaviruses. Expedition cruises travel through remote environments, combine passengers from many countries, operate with limited onboard medical capacity, and depend on the port state's willingness to receive ill travelers. The MV Hondius event required coordination among countries, the WHO, laboratories, port health authorities, clinical evacuation systems, and the cruise operator [19,21,22]. Several operational questions follow: Who has the authority to order isolation, disembarkation, or restrictions on onward travel? Which country is responsible for contact tracing after passengers return home? How are bodies managed respectfully when deaths occur at sea? How are communications handled when public fear exceeds the measured public risk? These are not purely technical questions; they are policy decisions involving law, ethics, logistics, and public trust [23,25]. The most defensible public health position is a precautionary but proportionate approach. WHO guidance advised controlled disembarkation, screening, personal protective equipment for medical teams, respirators for passengers and crew during disembarkation until assessment, active monitoring, and risk-based contact classification [21,22]. Such measures acknowledge uncertainty without implying that ANDV has become efficiently airborne or pandemic-like.

Policy decisions and governance

Policy decisions in hantavirus events should be structured around five questions: what is known, what is uncertain, what harms are plausible, what measures are proportionate, and how decisions will be revised when evidence changes. Table 1 summarizes practical decisions for cruise-associated or travel-associated clusters. The IHR framework is central because cruise-associated events are inherently cross-border [68]. However, IHR notification is only the start. Countries need interoperable legal tools for monitoring, quarantine, clinical transfer, occupational health protections, and privacy-preserving passenger manifests. Cruise operators should be required to maintain ship sanitation certificates, outbreak response plans, medical logs, cabin maps, ventilation and cleaning protocols, and prearranged medical evacuation agreements for rare high-consequence infections [22,23]. Equity should be explicit. Crew members may have less power than passengers to refuse unsafe work, report symptoms, or access follow-up care after repatriation. Policies must ensure wage protection, medical coverage, translation, culturally appropriate communication, and nonpunitive symptom reporting. Ethical response also requires dignified management of deceased persons, transparent criteria for movement restrictions, and appeal or review mechanisms when quarantine is prolonged (Table 6).

**Table 6:** Recommended policy positions for hantavirus outbreak management WHO: World Health Organization; ANDV: Andes virus Source: This table has been synthesized by the authors based on the data from [13,19-23,26,27,43,44,48-51,68]

Decision area	Recommended policy position	Rationale
Notification	Notify WHO and national International Health Regulations focal points promptly when severe respiratory illness, deaths, or multinational exposure occurs	Early notification enables coordinated tracing, laboratory referral, and consistent public messaging
Case definitions	Use staged categories (suspected, probable, confirmed, noncase); update definitions as evidence evolves	ANDV symptoms are nonspecific, and premature certainty can miss cases or overquarantine low-risk travelers
Disembarkation	Implement controlled, dignified, staggered disembarkation with symptom screening and medical evacuation pathways	Maintains public safety while avoiding indefinite confinement or stigmatization of passengers and crew
Contact management	Classify contacts by exposure intensity, cabin proximity, caregiving, intimate contact, healthcare exposure, and shared travel.	ANDV transmission is limited but plausible after close or prolonged exposure
Data sharing	Share deidentified clinical, epidemiological, and genomic data across jurisdictions under pre-agreed governance frameworks	Multinational clusters cannot be interpreted from fragmented national datasets alone
Risk communication	Communicate clearly: low public risk, severe individual risk, existing uncertainties, and reasons for precautionary measures	Builds trust and reduces both panic and complacency

Learning from COVID-19

The COVID-19 pandemic offers lessons, but not a simple template. SARS-CoV-2 spread efficiently through respiratory routes, including presymptomatic and asymptomatic transmission, whereas hantaviruses remain primarily zoonotic and ANDV transmission appears limited and context-dependent [18,24,56]. Treating the MV Hondius event as “another COVID” would be scientifically misleading. Ignoring COVID-era lessons would be equally unwise. First, closed conveyances can amplify uncertainty even when the pathogen has limited transmissibility. The Diamond Princess outbreak showed how cruise ships can become epidemiological laboratories and policy dilemmas, especially when jurisdiction, quarantine, and onboard care are unresolved [69,70]. For hantavirus, a cruise ship may not amplify transmission as efficiently as SARS-CoV-2, but it can amplify exposure classification, fear, and logistical complexity. Second, diagnostic readiness is a public good. COVID-19 demonstrated that testing bottlenecks distort case counts, delay isolation decisions, and undermine confidence [71,72]. For rare zoonoses, the challenge is not mass testing alone but rapidly connecting clinicians to reference laboratories and validated algorithms. Prepositioned primers, controls, proficiency testing, and specimen transport agreements should be part of rare-pathogen preparedness. Third, communication must be transparent about uncertainty. Over-reassurance can backfire when case counts rise; alarmist framing can create stigma and noncompliance. WHO’s phrasing that the event was serious while the wider public risk remained low is the correct balance for ANDV [19,20]. Messages should distinguish individual clinical severity from population transmissibility. Fourth, proportionality matters. COVID-19 normalized large-scale restrictions, but postpandemic fatigue makes blunt measures harder to sustain and ethically harder to justify. For ANDV, targeted active monitoring, risk-based quarantine, symptom education, and rapid clinical referral are more defensible than broad public restrictions [21,25]. Fifth, misinformation ecosystems now respond instantly to emerging outbreaks. Social media can exaggerate isolated clusters into imagined pandemics or dismiss severe disease as media hype. Public health agencies should publish timelines, case definitions, rationale for precautions, and updates in accessible language before rumors fill the gap.

One Health prevention and environmental control

One Health is not a slogan for hantaviruses; it is the core prevention strategy. Human cases are endpoints of ecological systems involving reservoirs, landscapes, climate, housing, waste, food storage, tourism, and health systems [9,10,38]. Effective prevention, therefore, requires integrated surveillance across human health, veterinary and wildlife sectors, environmental agencies, tourism operators, port health authorities, and local communities. For endemic regions, public health agencies should map reservoir distribution, seasonal rodent abundance, human case clusters, land-use changes, and climate anomalies [9,10,41]. For tourism operators, risk assessment should include pre-trip advisories, rodent-proof food storage, safe cleaning practices, avoidance of sleeping in rodent-infested structures, and protocols for febrile travelers returning from rural exposures [13,73].

Cruise and expedition operators need a specific One Health checklist before entering endemic regions: itinerary-based zoonotic risk assessment, waste and food control to avoid attracting rodents, inspection of excursion lodging and storage areas, training for guides and crew, medical escalation plans, passenger education, and rapid reporting procedures. These measures are less dramatic than quarantine, but they act earlier in the causal chain.

Future perspectives

Genomic Epidemiology and Source Attribution

Future outbreak investigations should integrate sequencing from patients, contacts, rodents, and environmental samples. Genomic data can clarify whether cases share a point-source exposure or represent sequential person-to-person spread. In a cruise-associated cluster, this distinction is operationally important. A genetically tight group of viruses among passengers with shared excursion histories may support a common environmental exposure, whereas viral sequences nested within plausible contact chains may strengthen evidence for interpersonal transmission. Genomics can also help distinguish an outbreak linked to a specific region from coincidental infections acquired in different places.

Sequencing should not be treated as a substitute for field epidemiology. Hantavirus genomes may show limited diversity over short transmission intervals, and missing samples from rodents, environments, or early human cases can distort reconstruction. Low diversity can make unrelated but geographically linked exposures appear connected, while incomplete sampling can make person-to-person transmission appear more linear than it truly was [12,49,59]. For these reasons, genomic interpretation should be integrated with incubation periods, symptom onset dates, travel itineraries, household contacts, caregiving episodes, clinical procedures, and environmental sampling.

Future systems should also strengthen the logistics of genomic response. This includes validated protocols for sample collection, cold-chain transport, data sharing, sequence quality control, metadata standards, and cross-border agreements for analysis. In an international travel cluster, sequences may be generated by laboratories in different countries using different platforms and reporting timelines. Preparedness therefore requires not only sequencing capacity but also governance for rapid comparison, transparent interpretation, and protection of patient privacy.

Source attribution should remain probabilistic rather than absolute. A well-supported conclusion may state that the balance of evidence favors a common environmental exposure, a close-contact transmission chain, or a mixed scenario. Overstating certainty can mislead policy; understating useful evidence can delay action. The future of hantavirus genomic epidemiology will depend on disciplined integration of molecular data with ecological investigation and human exposure reconstruction.

Clinical Prediction and Biomarkers

Rapid deterioration in HCPS/HPS creates a need for validated early-warning tools. Many patients initially present with fever, myalgia, headache, gastrointestinal symptoms, or malaise, which overlap with influenza, COVID-19, dengue, leptospirosis, rickettsioses, sepsis, and other travel-associated infections. The clinical challenge is to identify the small subset of febrile patients who are entering the cardiopulmonary phase before shock and respiratory failure become advanced. Delayed recognition can be especially consequential on ships, in rural hospitals, and in remote expedition settings where transfer to intensive care or ECMO-capable centers takes time.

Candidate markers include thrombocytopenia, hemoconcentration, leukocytosis, elevated lactate, renal dysfunction, inflammatory cytokines such as IL-6, angiopoietin imbalance, and viral RNA detection in multiple body fluids [50,56,58,61]. These markers are biologically plausible because severe disease is characterized by endothelial dysfunction, capillary leak, immune activation, and hemodynamic collapse. However, most candidate predictors require validation in larger, diverse cohorts and must be evaluated for real-world usability. A biomarker that performs well in a reference laboratory may not help a clinician on a vessel or in a small regional hospital unless sampling, transport, and turnaround time are feasible.

To operationalize predictive markers across diverse healthcare settings, clinicians need a tiered diagnostic framework that adapts to local laboratory capacity. In low‑resource environments such as shipboard infirmaries, rural clinics, or remote expedition posts, triage must rely on rapid hematological monitoring, with the key trigger being a serial complete blood count showing a sharp decline in platelets alongside rising hematocrit. When this biphasic signal appears during a nonspecific febrile prodrome in a traveler with compatible exposure history, it serves as a high‑sensitivity warning for early capillary leak, well before pulmonary edema or hemodynamic collapse becomes evident. Detection of these localized shifts should immediately prompt clinical stabilization, strict fluid restriction, and urgent evacuation to a tertiary center, while simultaneously securing blood or serum samples for transfer to reference laboratories. At this third tier, definitive confirmation and refined risk stratification can be achieved through quantitative RT‑PCR to track viral load dynamics and multiplex immunoassays to assess inflammatory mediators such as IL‑6 and angiopoietin. This structured approach ensures that simple, low‑cost field tests drive timely intervention, while advanced molecular assays are reserved for centralized confirmation, thereby preventing diagnostic paralysis in resource‑limited settings.

Future prediction models should combine clinical history, exposure risk, vital signs, routine laboratory tests, and specialized biomarkers. Practical tools might stratify patients into low, intermediate, and high risk for deterioration, guiding observation, transfer, isolation, and consultation with critical-care specialists. Such tools should be calibrated separately for different hantaviruses and regions because disease expression, healthcare access, and baseline differential diagnoses vary. They should also be evaluated prospectively rather than only retrospectively.

Equity is important in biomarker research. If prediction models depend on expensive assays unavailable in endemic rural areas, they may widen rather than reduce disparities. The most useful future tools will likely combine simple warning signs, routine laboratory abnormalities, and targeted molecular or immunological testing when available. For cruise and travel medicine, the priority is a decision framework that can be used early, before diagnostic certainty is complete.

Countermeasure Platforms

The rarity and geographic clustering of HCPS make traditional randomized trials difficult. Cases may appear unpredictably, often in remote areas, and severe disease can progress faster than research teams can assemble protocols. Preparedness should, therefore, include master protocols, adaptive trial designs, validated animal models, immunobridging assays, and regional biobanks established before outbreaks occur [51,60,65]. These platforms would allow investigators to evaluate interventions during sporadic cases or clusters without designing each study from the beginning.

Countermeasure development should include several complementary approaches. Vaccines may be most relevant for populations with recurrent occupational or regional risk, such as agricultural workers, forestry workers, military personnel, laboratory staff, and residents of highly endemic areas. Monoclonal antibodies or polyclonal antibody preparations may be useful for postexposure prophylaxis or early treatment if they can be delivered within a biologically meaningful window. Antivirals and host-directed therapies may be valuable if they reduce viral replication, endothelial dysfunction, immune-mediated injury, or progression to shock [60,62,63].

Feasibility should be evaluated as seriously as efficacy. A countermeasure requiring ultracold storage, late-stage intensive-care delivery, or complex high-containment handling may have limited impact in rural endemic settings. Conversely, a product with moderate efficacy but simple storage, rapid deployment, and a clear safety profile may be more useful in real outbreaks. Candidate vaccines and therapeutics should, therefore, be assessed for manufacturing scalability, dosing complexity, cold-chain requirements, safety monitoring, cost, and acceptability to affected communities.

For travel-associated events, countermeasure planning also raises policy questions. Authorities need criteria for who would receive postexposure prophylaxis if an effective product becomes available, how scarce doses would be allocated among passengers, crew, healthcare workers, household contacts, and endemic communities, and how informed consent would be managed during an emergency. These questions should be addressed before an outbreak because ad hoc allocation during a high-fear event can appear arbitrary and undermine trust.

Maritime Health Preparedness

Rare zoonoses should be integrated into ship outbreak plans that historically focused on norovirus, influenza, COVID-19, measles, and foodborne disease. Expedition vessels are especially relevant because they may visit remote ecological settings, use shore excursions, and operate far from advanced hospitals. A ship does not need to be the original site of rodent exposure to become the place where fever, respiratory symptoms, fear, and operational decisions converge.

Preparedness should begin before embarkation. Operators should conduct itinerary-specific risk assessments, review endemic zoonoses in planned regions, provide passenger education, train crew to recognize high-risk symptoms, inspect food and waste systems, and ensure that excursion partners follow rodent-control and safe-cleaning practices. Medical teams should know how to obtain public health consultation, collect and store specimens, arrange evacuation, and protect staff during examination or procedures. These steps are less visible than emergency quarantine, but they are more likely to prevent confusion when a rare disease is suspected.

Port authorities and cruise operators should practice tabletop exercises for high-consequence syndromes, including unclear transmissibility, deaths at sea, passenger refusal, media pressure, and multicountry repatriation [22,23]. Exercises should specify who has decision authority, how information flows between the ship, company, port state, flag state, national public health agencies, and WHO/IHR channels, and how passengers and crew receive updates. They should also include uncomfortable scenarios: inconclusive tests, delayed evacuation, crew members with limited labor protections, language barriers, medication needs during quarantine, and families demanding immediate repatriation.

Maritime preparedness must be humane as well as technical. Isolation areas should preserve dignity, food and medication access should be guaranteed, communication with families should be supported, and crew members should not be treated as expendable operational assets. To translate these ethical imperatives into practice, port states and maritime authorities must embed equity protections directly into standard operating procedures rather than relying on improvised emergency measures. Health policies should require structured mental health support for passengers and crew in isolation, including daily access to remote counseling, natural light, and secure, unmonitored communication with families, since confinement in small, windowless cabins greatly increases psychiatric risk. At the same time, maritime labor rights must be legally safeguarded during zoonotic crises, ensuring that crew members under isolation or quarantine receive full wage protection, nonpunitive medical leave, and immunity from contract termination or retaliatory repatriation. When the crew is tasked with high‑risk duties such as medical triage, disinfecting rodent‑contaminated spaces, or direct caregiving, they must be guaranteed hazard pay, comprehensive medical coverage, and appropriate protective equipment. Embedding these human rights and labor equity standards into the IHR and maritime inspection frameworks ensures that public safety measures do not devolve into coercive practices that disproportionately burden a transient workforce [22,23]. The COVID-19 cruise experience showed how quickly ships can become symbols of fear and policy failure. Hantavirus planning should avoid repeating that pattern by combining proportionate precautions with transparent communication and practical support [24].

Research priorities

Key research priorities include determining the infectious dose and dominant ANDV transmission routes; identifying the timing and magnitude of infectious shedding; measuring asymptomatic or paucisymptomatic infection; quantifying the effectiveness of contact precautions; validating rapid molecular and serologic assays; evaluating ECMO referral thresholds; developing therapeutics and vaccines; and modeling how climate and land use alter future spillover risk [10,18,50,69,73]. These priorities are interconnected rather than independent. For example, better knowledge of shedding informs contact definitions, which shape quarantine policy, which affects the feasibility of clinical follow-up and research recruitment.

Transmission research should focus on the conditions that make ANDV exceptional. Studies should clarify whether infectious virus in saliva, nasopharyngeal specimens, urine, or gingival crevicular fluid translates into a meaningful risk of transmission, and whether that risk is concentrated during particular illness stages [50]. Household and caregiving studies should collect detailed exposure data so that intimate contact, shared environmental exposure, healthcare procedures, and social proximity can be separated more clearly. Animal models should be used to test mechanisms, but human epidemiology remains essential for estimating real-world risk.

Clinical research should prioritize early recognition and severity prediction. Prospective cohorts in endemic regions could collect standardized data on symptoms, exposure history, complete blood count, metabolic markers, inflammatory mediators, viral load, imaging, oxygen requirement, shock, renal dysfunction, and outcomes. Harmonized case report forms would allow small outbreaks to contribute to cumulative evidence. Because severe disease is uncommon, international collaboration is necessary to generate sample sizes large enough for reliable prediction models and treatment evaluation.

Diagnostic research should emphasize speed, accessibility, and interpretation. Rapid molecular or serologic assays could transform shipboard and rural response if they are accurate early in illness, easy to transport, and usable outside major reference laboratories. However, false positives and false negatives can both have serious consequences in a high-anxiety travel cluster. Validation should, therefore, include different illness stages, virus genotypes, specimen types, and operational settings.

Ecological and policy research should be integrated with biomedical priorities. Climate variability, land conversion, tourism expansion, housing quality, and occupational exposure can alter spillover risk before clinicians ever see a patient [9-11]. Future work should evaluate which prevention investments reduce human disease most effectively: rodent-proof housing, community education, occupational protections, safer tourism infrastructure, environmental monitoring, or targeted vaccination if vaccines become available. The strongest research agenda will connect ecology, clinical care, and governance rather than treating them as separate fields.

Selected outbreaks and evidence milestones

The management of modern travel‑associated pathogen clusters is grounded in decades of outbreak investigations and virological discoveries rather than occurring in isolation. By tracing key milestones in hantavirus epidemiology since the late 20th century, clinicians and public health authorities can see how scientific consensus evolved from viewing hantaviruses solely as localized, environmentally acquired zoonoses to recognizing the unique human‑to‑human transmission dynamics of certain genotypes. Table 4 presents these milestones chronologically, illustrating how events ranging from the identification of SNV in North America to severe localized outbreaks in South America have shaped contemporary global health governance, informed active contact‑tracing protocols, and strengthened maritime biosecurity preparedness (Table 7).

**Table 7:** Historical milestones in hantavirus outbreaks and preparedness relevance ANDV: Andes virus; RNA: ribonucleic acid

Study	Location/event	Key findings	Preparedness relevance
World Health Organization [19]	MV Hondius (cruise ship)	A multinational, travel-associated ANDV cluster that triggered rapid cross-border International Health Regulations coordination	Served as an operational biosecurity stress test for maritime health governance, contact tracing across jurisdictions, and post-COVID-19 risk communication
Coelho et al. [49]	Argentina and Chile	Successful isolation and complete virological characterization of contemporary, circulating ANDV strains	Provided an updated molecular platform for real-time diagnostic assay validation, source attribution, and therapeutic testing
Barrera et al. [59]			
Ferrés et al. [50]	Chile (prospective cohort)	Infectious ANDV RNA and live virus detected across multiple peripheral body fluids (saliva, urine, nasopharyngeal fluid) during the acute phase	Strengthened the biological plausibility for close-contact interpersonal transmission and informed standard droplet precautions
Toledo et al. [18]	South America (systematic review)	Systematic evaluation of global human-to-human transmission datasets, noting potential vulnerability to coexposure bias	Refined clinical risk assessment paradigms, showing that true interpersonal spread is low, inefficient, and highly context-dependent
Martinez et al. [17]	Epuyén, Argentina	Combined field epidemiology and genomic tracking to trace a 34-case person-to-person cluster showing clear superspreading dynamics	Demonstrated the critical value of genomic epidemiology in reconstructing complex transmission chains and enforcing targeted containment
Martinez-Valdebenito et al. [48]	Southern Chile	Confirmed and detailed sequential cases of household and nosocomial ANDV transmission	Reinforced the necessity of rapid healthcare infection prevention, strict isolation protocols, and close-contact exposure monitoring
Ferres et al. [16]	Chile	Conducted prospective evaluations of household contacts exposed to patients with severe cardiopulmonary syndrome	Justified the deployment of active symptom tracking and home-monitoring for intimate or long-term household partners
Martinez et al. [15]	Argentina	Expanded field epidemiological confirmation of sequential human-to-human transmission events outside of animal environments	Established formal case and contact management strategies for individuals with close, prolonged, noncasual exposure
Padula et al. [14]	Argentina	Discovered clear molecular and genetic evidence supporting the person-to-person transmission of ANDV	Formally introduced ANDV as a critical, unique exception to the established rule that hantaviruses are strictly zoonotic threats
Nichol et al. [28]	Four Corners, United States	Identification and clinical characterization of Sin Nombre virus following a lethal cluster of acute respiratory illness	Established initial global recognition of hantavirus pulmonary/cardiopulmonary syndrome and modernized surveillance in the Americas
Duchin et al. [42]			

Discussion

The MV Hondius cluster illustrates that rare zoonoses can create global coordination challenges without becoming pandemics. The central risk is not that hantavirus has acquired SARS-CoV-2-like transmissibility; the central risk is that a severe, diagnostically unfamiliar infection can move through travel networks faster than local systems can classify exposure, test samples, transfer patients, and communicate clearly. This distinction matters because preparedness must be scaled to the hazard. A response that is too weak may miss cases and delay treatment; a response that is too sweeping may create avoidable social, ethical, economic, and psychological harm.

The first implication is clinical. HCPS/HPS often begins with fever, myalgia, headache, gastrointestinal symptoms, and malaise that overlap with influenza, COVID-19, dengue, leptospirosis, rickettsioses, sepsis, and other travel-associated infections [6,37]. By the time pulmonary edema, shock, severe thrombocytopenia, or hemoconcentration is obvious, clinical deterioration may be rapid. Clinicians evaluating travelers from endemic rural or wilderness areas should therefore ask specifically about rodent exposure, cabin or campsite conditions, cleaning of enclosed spaces, agricultural or forestry activity, expedition excursions, sick contacts, and cruise or group travel. Early suspicion can accelerate reference laboratory testing, infection prevention, transfer to centers with critical-care capacity, and consultation with public health authorities.

The second implication is diagnostic and operational. Hantavirus diagnosis depends on compatible clinical illness, exposure history, serology, molecular testing, and specialist interpretation. In a ship-linked event, these requirements are difficult to meet because patients may disembark in different countries, samples may be collected at different illness stages, and case definitions may evolve as evidence accumulates. A practical response requires standardized line lists, specimen referral pathways, agreed reporting intervals, and transparent rules for classifying confirmed, probable, possible, and excluded cases. Genomic epidemiology can strengthen inference about transmission chains, but it cannot replace careful reconstruction of exposures, timing, symptoms, and contact patterns [17,18,22].

The third implication concerns transmission messaging. ANDV should be described neither as a routine rodent exposure with no interpersonal relevance nor as a highly transmissible respiratory virus. The most accurate public message is that most hantavirus infections result from contact with infected rodents or contaminated environments, while ANDV has shown limited person-to-person transmission after close and prolonged exposure [14-17,50]. This framing supports targeted contact monitoring, appropriate infection prevention in healthcare and caregiving settings, and avoidance of stigmatizing passengers, crew, or communities associated with the event. It also helps explain why global public risk may remain low even when individual cases are severe.

The fourth implication is that outbreak investigation must integrate clinical medicine with environmental epidemiology. A cruise-associated cluster cannot be interpreted only by studying the ship. Investigators must reconstruct pre-embarkation travel, shore excursions, lodging, food storage areas, waste handling, rodent sightings, cleaning activities, passenger social networks, cabin assignments, caregiving relationships, medical encounters, and post-disembarkation travel. If an onshore exposure seeded multiple infections, shipboard control measures alone will not explain the event. If close-contact transmission occurred on board or after disembarkation, environmental sampling alone will be insufficient. A One Health investigation should therefore connect rodent ecology, human movement, clinical timelines, and laboratory data.

The fifth implication is maritime preparedness. Cruise operators and port health authorities should not design plans only for common outbreaks such as norovirus, influenza, COVID-19, measles, or foodborne illness. They also need decision frameworks for rare but severe syndromes in which transmissibility, incubation, diagnostic certainty, and medical evacuation needs are initially unclear. Such frameworks should specify how to isolate an ill person humanely, protect healthcare and service staff, preserve essential ship operations, communicate with passengers and families, coordinate with flag states and port states, and avoid abandoning crew members who may have fewer resources than passengers [22,23]. Preparedness is not simply a stockpile of masks or disinfectants; it is a rehearsed governance process for uncertainty.

The sixth implication is ethical. Quarantine, movement restriction, and contact monitoring may be justified when evidence supports a plausible risk of severe disease and onward transmission, but these measures must be proportionate, time-limited, evidence-based, and accompanied by practical support [21,25]. Passengers and crew require food, medications, language-accessible information, mental health support, communication with families, salary and employment protections where relevant, and clear procedures for appeal or review. Ethical legitimacy also depends on reciprocity: people asked to restrict movement for public benefit should not be left to bear the burden alone.

The seventh implication is communication. COVID-19 changed the social environment in which every outbreak unfolds. Publics are more alert to transmission risks but also more fatigued, polarized, and vulnerable to misinformation [24,25]. Hantavirus communication must therefore be concise, repeated, and explicit about uncertainty. Authorities should state what is known, what is not yet known, what is being done to reduce uncertainty, and what actions are recommended for different audiences. For the general public, the message should emphasize that severe disease is possible, but that routine risk remains low outside defined exposure or contact groups. For contacts, the message should emphasize symptom monitoring, rapid reporting, and access to care rather than blame.

The eighth implication is equity. Rare outbreaks often expose differences in power between tourists, crew, local communities, port authorities, and endemic-region residents. Cruise passengers may receive international media attention and rapid repatriation, while crew members and local workers may face employment insecurity, stigma, or less visible medical needs. Similarly, endemic rural communities may be portrayed as sources of danger rather than as partners in surveillance and prevention. One Health preparedness should include community engagement, occupational protection, rodent-safe housing and storage practices, and sustained investment in local diagnostic and clinical capacity rather than episodic attention only when international travelers are affected.

The ninth implication is research readiness. The rarity of HCPS/HPS makes conventional trials difficult, but the consequences of severe disease justify advance planning. Adaptive protocols, regional clinical networks, standardized severity scores, biobanks, genomic sequencing capacity, and harmonized outcome measures should be prepared before outbreaks occur [51,60,65]. Research priorities include defining the timing and magnitude of infectious shedding, clarifying the role of saliva and other body fluids, validating rapid diagnostics, identifying early biomarkers of deterioration, optimizing ECMO referral thresholds, and evaluating vaccines, monoclonal antibodies, antivirals, and host-directed therapies.

Finally, the MV Hondius event should be interpreted as a warning against both complacency and exaggeration. Complacency would ignore the high case-fatality potential of HCPS/HPS, the documented exception of ANDV person-to-person transmission, and the operational difficulty of managing severe illness during travel. Exaggeration would imply that a rodent-borne zoonosis with limited close-contact transmission behaves like a pandemic respiratory virus. The appropriate middle path is disciplined preparedness: early clinical recognition, rapid laboratory confirmation, careful contact assessment, humane risk-based restrictions, international coordination, ecological investigation, and communication that is precautionary without being alarmist.

Limitations

This manuscript is presented as a narrative review rather than a systematic review. It synthesizes peer‑reviewed literature and contemporaneous official guidance but does not employ duplicate screening, formal risk‑of‑bias assessment, or meta‑analysis. Outbreak data from 2026 remain time‑sensitive and subject to change after manuscript preparation. Accordingly, cruise‑associated case counts, classifications, transmission chains, and genomic interpretations should be verified and updated.

## Conclusions

Hantaviruses continue to pose a persistent One Health challenge at the intersection of wildlife reservoirs, evolving land-use patterns, and human mobility. The 2026 MV Hondius outbreak functioned less as a pandemic precursor than as an operational biosecurity stress test, illustrating how modern travel networks can rapidly amplify a rare terrestrial zoonosis into a complex, multijurisdictional public health event. While the ecological constraints of hantaviruses distinguish them from respiratory pathogens capable of sustained global transmission, the ANDV remains exceptional in its context-dependent potential for close-contact spread, necessitating tailored containment strategies. For both shipboard medical teams and rural frontline clinics, a tiered diagnostic approach, anchored in simple, field-ready indicators such as exposure history combined with thrombocytopenia and hemoconcentration, remains critical for triggering early fluid management and urgent evacuation before hemodynamic collapse.

Looking ahead, international health governance must move beyond reactive containment toward proactive, cross-border preparedness platforms. This requires harmonization under the IHR, to codify maritime labor equity, ensure wage protection for isolated crew, and establish interoperable genomic surveillance networks. Research priorities should focus on validating rapid point-of-care assays, identifying early biomarkers of endothelial dysfunction, and advancing scalable countermeasure pipelines under the animal-rule framework. Ultimately, managing travel-associated zoonotic risks demands calibrated risk communication, avoiding both the complacency of underestimating clinical severity and the alarmism of miscasting localized spillovers as imminent pandemic threats.
